# Oral Health, Loneliness and Social Isolation. A Systematic Review and Meta-Analysis

**DOI:** 10.1007/s12603-022-1806-8

**Published:** 2022-06-04

**Authors:** André Hajek, B. Kretzler, H.-H. König

**Affiliations:** Department of Health Economics and Health Services Research, University Medical Center Hamburg-Eppendorf, Hamburg Center for Health Economics, 20246 Hamburg, Germany

**Keywords:** Loneliness, social exclusion, social isolation, oral health, oral health-related quality of life

## Abstract

**Objectives:**

Thus far, some empirical studies have investigated the association between oral health and loneliness as well as social isolation. However, a systematic review and meta-analysis is lacking synthesizing this evidence. Hence, our purpose was to close this knowledge gap.

**Design:**

Systematic review and meta-analysis.

**Setting and Participants:**

Observational studies examining the association between oral health and loneliness or social isolation were included. Disease-specific samples were excluded.

**Methods:**

We searched three electronic databases (PubMed, PsycINFO, CINAHL), and did an additional hand search. Data extraction covered methods, sample characteristics and main findings. To evaluate study quality/risk of bias, the NIH tool was used. Study selection, data extraction and assessment of study quality were each conducted by two reviewers.

**Results:**

Seven studies were included in our current work. Several cross-sectional studies and one longitudinal study reported an association between poorer oral health and higher loneliness as well as higher social isolation. The quality of the studies was mostly fair, with two studies of high quality. The pooled OR was 1.47 (95% CI 1.24–1.75) among the studies with adult samples.

**Conclusion:**

Most of the included studies demonstrated an association between oral health and loneliness or social isolation. There is a lack of high quality studies on these associations; in particular, future studies should use longitudinal data to clarify the directionality between oral health and loneliness or social isolation. Prospero registration number CRD42021268116.

**Electronic Supplementary Material:**

Supplementary material is available for this article at 10.1007/s12603-022-1806-8 and is accessible for authorized users.

## Introduction

**T**he awareness of loneliness and social isolation markedly increased in the general public and even in various research areas in the past few years. This is particular true in times of the Covid-19 pandemic where social distancing is frequent. The importance of loneliness (perceived discrepancy between actual and desired social contacts (1)) and social isolation (feeling that one is left out from society (2)) can also be stressed because they are associated with chronic conditions and longevity (3, 4).

A number of studies have examined the factors contributing to loneliness and isolation covering socioeconomic factors, factors related to lifestyle, psychosocial factors and factors related to health (5, 6). Some studies have also examined the association between oral health and loneliness as well as social isolation (7–10). However, thus far, a systematic review and meta-analysis is lacking systematically synthesizing this evidence. Hence, the objective of this systematic review and meta-analysis was to synthesize existing research on the association between oral health, loneliness and social isolation. This knowledge may be beneficial in characterizing individuals at risk for loneliness and social isolation. In turn, from a public health perspective this is of great importance because these factors can contribute to successful ageing, general health, and mortality (3, 4, 11). Additionally, it should be noted that this knowledge is also important because oral health is modifiable (12). Such knowledge is also important for dentists. Moreover, our work may identify potential gaps in our knowledge and may therefore encourage future research.

## Materials and Methods

Our work was conducted in line with the Preferred Reporting Items for Systematic Reviews and Meta-Analysis guidelines (13). It is registered with the International Prospective Register of Systematic Reviews (PROSPERO, registration number: CRD42021268116).

All steps (search, data extraction and quality assessment) were independently conducted by two individuals (AH, BK). Discussion was used to achieve a consensus in case of disagreement (if required: a third party (HHK) was contacted).

### Search strategy and selection criteria

Three databases (PubMed, PsycINFO, CINAHL) were searched in October 2021. The search query for PubMed is shown in Supplementary Table 1. For example, the search strategy includes terms such as “oral health*”, “lonel*” or “social isolation”. A two-step process was used for assessment of inclusion/exclusion (1: title/abstract screening and 2: full-text screening). Moreover, we conducted a hand search.

**Table 1:** Study overview

First Author (Year)	Country	Assessment of loneliness, and social Isolation	Assessment of oral health	Study Type	Sample Characteristics: Sample Descriptions Sample size Age Females in total sample	Results	Overall quality judgment
Delgado-Angulo (2009)	Peru	assessed through eleven items regarding distributional and material, relational and participatory, and longterm perspectives of social isolation	clinical investigation regarding the number of decayed, missing and filled teeth	cross-sectional	children from Lima living with their families n=90 Age: 12 Females in total sample: 53.3%	Binary logistic regression revealed that social isolation was not significantly associated with dental caries (OR: 1.79, 95% CI: 0.94–3.43).	Fair
Koyama (2021)	Japan, United Kingdom	Social Isolation Score (five items)	self-reported number of remaining teeth, use of dentures	cross-sectional	English Longitudinal Study of Ageing, Japan Gerontological Evaluation Study n=124,153 Age: M: 73.8 SD: 6.1 ≥65 Females in total sample: 51.0%	According to ordered logistic regression, both denture use and a decreased number of teeth were related to increased odds of social isolation (e.g., no teeth vs. 20+: OR: 1.21, 95% CI: 1.15–1.27).	Good
Lundgren (1995)	Sweden	feeling lonely (dichotomous)	self-reported dental state (four categories), symptoms from teeth or dentures and/or mouth dryness (rated on a three-point-scale) and chewing ability (rated on a three-point scale)	cross-sectional	randomly selected participants from the Gerontological and Geriatric Population Studies n=374 Age: 87–88 Females in total sample: 70.3%	Feelings of loneliness were correlated to symptoms from teeth and/or dentures (r=0.18, p<.01).	Fair
Monteiro da Silva (1996)	United Kingdom	UCLA Loneliness Scale (20 items)	clinical diagnosis of rapidly progressive periodontitis (dichotomous) or routine chronic adult periodontitis (dichotomous)	cross-sectional	patients from a dental hospital n=150 Age: M: 40.6 SD: 6.9 Females in total sample: 66%	Loneliness was more prevalent in individuals suffering from rapidly progressive periodontitis (38.5%) than in the control group (32.0%) (p=.003).	Fair
Olofsson (2018)	Finland, Sweden	feeling lonely (dichotomous), being socially isolated (never having any contact with neighbours or friends, dichotomous)	self-reported edentulism (not mainly having one’s own permanent teeth less, dichotomous)	cross-sectional	Gerontological Regional Database Study n=6,099 Age: 65: 40,4% 70: 24,9% 75: 19,8% 80: 14,9% Females in total sample: 53.1%	With respect to logistic regression, higher social isolation was associated with being edentulous (OR: 1.52, 95% CI: 1.17–1.98), whereas experiencing loneliness was not associated with being edentulous (OR: 1.11, 95% CI: 0.85–1.44)	Fair
Rouxel (2017)	United Kingdom	UCLA Loneliness Scale (three items)	Oral Impact on Daily Performances (ten items)	cross-sectional and longitudinal (two waves from 2006 to 2011)	English Longitudinal Study of Ageing n=6299 Age: 50–64: 54,3% 65–74: 26,2% ≥ 75: 19,5% Females in total sample: 52.4%	Logistic regressions (cross-sectional) showed an association between lower oral health and higher loneliness (OR: 1.48, 95% CI; 1.16–1.88) Regarding multinomial logistic regression (longitudinal analysis), incident oral impact was significantly associated with the likelihood of becoming lonely (OR: 1.56, 95% CI: 1.09–2.25).	Good
Singh (2020)	India	Patients Reported Outcomes Measurement Information System’s social isolation 8a, short form	Clinical assessment: decayed, missing, and filled tooth index; periodontal disease; and edentulousness (WHO-criteria)	Cross-sectional	Department of Dentistry, All India Institute of Medical Sciences; 3 Altenheime in Bhopal (Indien, 2017) n=421 Age: 60–70: 70,3 % 71–80: 23,0 % 81–100: 6,7 % Females in total sample: 36.3%	Loneliness was significantly associated with decayed, missing, and filled tooth index scores ((OR 1,86; 95% CI 1,38–3,20), periodontal disease (OR 1,29; 95% CI 1,13–3,11) and edentulousness (OR 2,37; 95% CI 1,18–3,58).	Fair

Inclusion criteria were as follows:

- Cross-sectional and longitudinal observational studies investigating the association between oral health and loneliness or social isolation
- Studies adequately quantifying key variables
- Studies published in peer-reviewed journals (German or English language).

Contrary, studies were excluded when they exclusively used samples with a specific disorder (e.g., individuals with anxiety). Studies based on samples exclusively restricted to individuals with a specific illness were excluded because we were interested in studies which are widely generalizable and not limited to specific illnesses. However, it is worth noting that we included observational studies which also include but are not limited to individuals with specific illnesses.

No restrictions were applied regarding gender, ethnicity or country. Furthermore, samples of any age category were included. We did a pretest (sample of 100 titles/abstracts) before final eligibility criteria. Nevertheless, eligibility criteria did not change.

### Data extraction and analysis

One reviewer (BK) conducted the data extraction. It was cross-checked by a second reviewer (AH). Extraction of the data included study design, operationalization of key variables, describing the sample and main findings (association with oral health and loneliness/social isolation). In the results section, the key findings are displayed separated by loneliness and social isolation.

### Quality assessment

The NIH Quality Assessment Tool for Observational Cohort and Cross-Sectional Studies (14) was used to evaluate the quality of the studies included. It is a well-known and widely-established tool when assessing the quality of observational studies.

### Meta-analysis

It should be noted that we additionally conducted a random-effects meta-analysis. In our main model, we only excluded one study referring to children (and thus focused on adult samples). In additional analysis, we restricted our meta-analysis to studies which were rated as “good”. In line with given recommendations, heterogeneity between studies was assessed using the I^2^ statistic (I^2^ values between 25% to 50%: low; 50% to 75%: moderate; 75% or more: high heterogeneity (Higgins et al., 2003). Stata 16.1 was used to conduct meta-analysis.

## Results

### Overview: included studies

**Figure 1:**
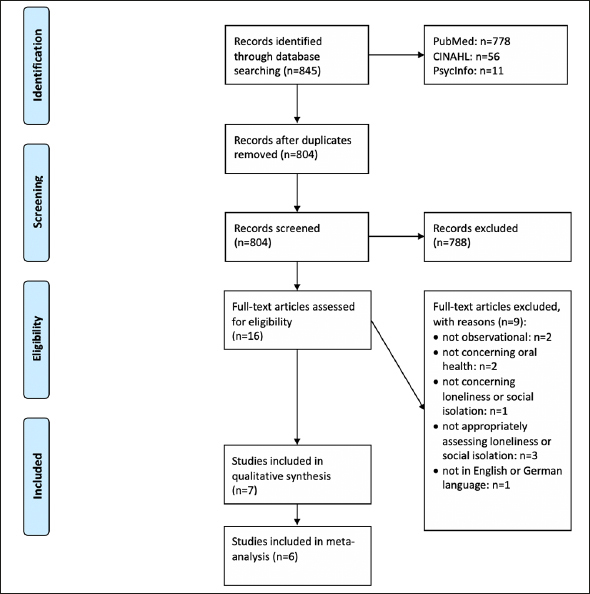
Flow chart From: Moher D, Liberati A, Tetzlaff J, Altman DG, The PRISMA Group (2009). Preferred Reporting Items for Systematic Reviews and Meta-Analyses: The PRISMA Statement. PLoS Med 6(7): e1000097. doi:10.1371/journal.pmed1000097

The selection process of the studies is shown in Figure 1 (flow chart (15)). Seven studies satisfied our eligibility criteria and were thus included in our current work (7–10, 16–18).

An overview is provided in Table 1 (including key findings). If possible, results of adjusted regressions are shown.

Data came from Europe (n=6 samples, with: United Kingdom, n=3; Sweden, n=2; Finland, n=1), Asia (Japan, n=1; India, n=1) and South America (Peru, n=1).

One study used data from both United Kingdom and Japan (7) and a second study used data from Finland and Sweden (8). Six out of the seven studies solely used cross-sectional data. Only one study used both cross-sectional and longitudinal data (two waves from 2006 and 2011) (9). While the studies from Europe (and Japan) mainly used data from large, representative samples, the other studies used more specific samples (e.g., patients from a dental hospital (18)). The sample size varied from 90 (16) to 124,153 individuals (7). There was some variety in the assessment of loneliness or social isolation. For example, two studies used different versions of the UCLA tools to quantify loneliness, whereas two studies used a single-item to quantify loneliness. Similarly, there was a large variety in the operationalization of oral health (e.g., self-reported number of remaining teeth or the tool “Oral Impact on Daily Performances”). Five studies examined older adults, whereas one study examined children aged 12 years (16) and one study examined middle-aged adults (18). The proportion of women ranged from 36% to 70% (with most of the studies having 50% to 60% female participants). Only one study examined the association between oral health and both loneliness and social isolation (8). Further details are given in Table 1.

### Oral health and loneliness

In sum, n=5 studies examined the association between oral health and loneliness (8–10, 17, 18). Four of those studies were solely cross-sectional (8, 10, 17, 18), whereas one study performed both cross-sectional and longitudinal analyses (9). Three out of the four cross-sectional studies showed an association between lower oral health and higher loneliness, whereas Olofsson et al. did not identify such an association in multiple regression analysis (also adjusting for social isolation) (8). It should be noted that Olofsson found a significant bivariate association between lower oral health and higher loneliness (8). Only Rouxel et al. (9) treated loneliness as dependent variable, whereas the other studies using regression analysis treated oral health as dependent variable. They showed that lower oral health was associated with higher loneliness both cross-sectionally and longitudinally (9). None of the studies examined gender differences in the association between oral health and loneliness.

### Oral health and social isolation

In sum, n=3 cross-sectional studies examined the association between oral health and social isolation (7, 8, 16). Two out of the three studies found an association between low oral health and high social isolation among older adults (7, 8), whereas one study did not find such an association among children in Peru (16). Two studies treated social isolation as independent variable (8, 16), whereas one study treated social isolation as outcome measure (7). None of the three studies examined gender differences in the association between oral health and social isolation. Longitudinal studies are missing investigating the association between oral health and social isolation.

### Quality assessment

In Table 2, the quality assessment is shown. While some criteria were fulfilled by all studies (e.g., objective clearly stated), other criteria were rarely satisfied (e.g., sufficient response rate). The general quality of the included studies was mainly fair. More precisely, five studies were rated as ‘fair’, whereas two studies were rated as ‘good’. None of the studies were rated as ‘poor’.

**Table 2:** Quality assessment

Questions	Studies						
	Delgado-Angulo (2009)	Koyama (2021)	Lundgren (1995)	Monteiro da Silva (1996)	Olofsson (2018)	Rouxel (2017)	Singh (2020)
1. Was the research question or objective in this paper clearly stated?	yes	yes	yes	yes	yes	yes	yes
2. Was the study population clearly specified and defined?	yes	yes	yes	yes	yes	yes	yes
3. Was the participation rate of eligible persons at least 50%?	yes (98.9%)	not reported	yes (82%)	not reported	yes (63.9%)	not reported	not reported
4. Were all the subjects selected or recruited from the same or similar populations (including the same time period)? Were inclusion and exclusion criteria for being in the study prespecified and applied uniformly to all participants?	yes	yes	yes	yes	yes	yes	yes
5. Was a sample size justification, power description, or variance and effect estimates provided?	yes	no	no	no	no	no	yes
6. For the analyses in this paper, were the exposure(s) of interest measured prior to the outcome(s) being measured? (if not prospective should be answered as ‘no’, even is exposure predated outcome)	no (cross-sectional)	no (cross-sectional)	no (cross-sectional)	no (cross-sectional)	no (cross-sectional)	No (simultaneously)	no (cross-sectional)
7. Was the timeframe sufficient so that one could reasonably expect to see an association between exposure and outcome if it existed?	no (cross-sectional)	no (cross-sectional)	no (cross-sectional)	no (cross-sectional)	no (cross-sectional)	yes	no (cross-sectional)
8. For exposures that can vary in amount or level, did the study examine different levels of the exposure as related to the outcome (e.g., categories of exposure, or exposure measured as continuous variable)?	continous	categorical	dichotomous	dichotomous	dichotomous	dichotomous	continous
9. Were the exposure measures (independent variables) clearly defined, valid, reliable, and implemented consistently across all study participants?	yes	yes	yes	yes	yes	yes	yes
10. Was the exposure(s) assessed more than once over time?	no	no	no	no	no	yes	no
11. Were the outcome measures (dependent variables) clearly defined, valid, reliable, and implemented consistently across all study participants?	yes	yes	yes	yes	yes	yes	yes
12. Was loss to follow-up after baseline 20% or less?	not applicable	not applicable	not applicable	not applicable	not applicable	no	not applicable
13. Were key potential confounding variables measured and adjusted statistically for their impact on the relationship between exposure(s) and outcome(s)?	yes	yes	no	no	yes	yes	yes
Overall quality judgement	Fair	Good	Fair	Fair	Fair	Good	Fair

### Meta-analysis

Our meta-analysis (based on six studies which were conducted among adult samples) showed that the pooled OR was 1.47 (95% CI: 1.24–1.75); with social isolation as outcome: OR = 1.31 (1.06–1.61), with loneliness as outcome: OR = 1.63 (1.36–1.95). We identified moderate heterogeneity across these studies (P=60.3%). Please see Supplementary File 2 for further details. Based on the two studies which were rated as ‘good’, an additional meta-analysis was conducted. Again, moderate heterogeneity (I^2^ = 61.0%) across these two studies were identified. The pooled OR was 1.29 (95% CI: 1.07–1.55). Please see Supplementary File 3 for further details.

## Discussion

The purpose of our systematic review was to give an overview of empirical studies investigating the association between oral health and loneliness as well as social isolation. In total, seven studies were included in our review. Several cross-sectional studies and one longitudinal study reported an association between poorer oral health and higher loneliness as well as higher social isolation. The quality of the studies was mostly fair, with two studies of high quality. Moreover, a metaanalysis was conducted. The pooled OR was 1.47 (95% CI: 1.24–1.75) among the studies with adult samples.

The question arises why oral health is linked to loneliness and social isolation. A possible explanation may be that oral health is positively associated with mental health (19) which in turn is associated with loneliness and social isolation (20). Furthermore, previous research showed an associated between oral health and the status of being homebound - which could contribute to isolation or loneliness (21). Additionally, feelings of shame and feelings of stigmatization because of low oral health (which may be seen as a proxy for a low socio-economic status) may diminish the self-worth and contentment of individuals (22). Thus, individuals may also report feelings of isolation and loneliness (23). Other ways to explain such an association between oral health and loneliness and social isolation are as follows: Individuals with a low oral health may think that they are worse off (in terms of health) compared to other individuals in their age group. Such negative health comparisons may result in isolation - as previously shown (24). Another explanation may be that a low oral health may decrease overall health and well-being (25) which in turn can affect loneliness and isolation (26).

It should be noted that the comparability between the studies is somewhat restricted, e.g. due to differences in the age groups, and the heterogeneity in the assessment of the main variables (e.g., assessment of loneliness or oral health). In contrast, some factors are comparable between the studies: Most of the studies used a cross-sectional design. Furthermore, most of the existing studies used data from older adults living in European countries.

Several gaps in our knowledge were identified in our work which may guide future research. First, longitudinal studies are urgently needed to clarify the directionality between oral health and loneliness as well as social isolation. Second, more studies are required to clarify the association between oral health and loneliness or social isolation among children/adolescents as well as among young and middle-aged adults. Third, studies from other regions are needed (e.g., North America or Africa). Fourth, moderating (e.g., gender) and mediating factors (e.g., general self-esteem) in the association between oral health and loneliness and social isolation should be further studied.

Some strengths and limitations of our current work are worth noting. We performed the first systematic review and meta-analysis examining the association between oral health and loneliness as well as social isolation. Moreover, key steps (study selection, data extraction and evaluation of the quality) were performed by two reviewers. Additionally, a meta-analysis was conducted. While the decision to include only articles published in peer-reviewed journals ensures a certain quality of the studies, it also excludes potential studies of interest. Similarly, due to language restrictions, some studies of interest might be excluded.

## Conclusions

Most of the included studies demonstrated an association between oral health and loneliness as well as social isolation. There is a lack of high quality studies on these associations; in particular, future studies should use longitudinal data to clarify the directionality between oral health and loneliness or social isolation.

## Electronic supplementary material

**Supplementary Table 1**. Search strategy (PubMed).

Supplementary material, approximately 95.0 KB.

Supplementary material, approximately 92.9 KB.

## Notes

*Ethics:* Not applicable.
