# Supplementary material for: Oral Health, Loneliness and Social Isolation. A Systematic Review and Meta-Analysis
Source: J Nutr Health Aging. 2022 Jun 4;26(7):675–80. doi: 10.1007/s12603-022-1806-8 (PMC9166168; doi:10.1007/s12603-022-1806-8)
Supplement: Supplementary file 1 — Supplementary Table 1. Search strategy (PubMed). [file 12603_2022_1806_MOESM1_ESM.docx]

**Supplementary Table 1.** Search strategy (PubMed).

| #1 | Oral health* |
| --- | --- |
| #2 | Dental health* |
| #3 | #1 OR #2 |
| #4 | Lonel* |
| #5 | Social isolation |
| #6 | Social exclusion |
| #7 | #4 OR #5 OR #6 |
| #8 | #3 AND #7 |
