# Supplementary figures and images for: Oral Health, Loneliness and Social Isolation. A Systematic Review and Meta-Analysis
Source: J Nutr Health Aging. 2022 Jun 4;26(7):675–80. doi: 10.1007/s12603-022-1806-8 (PMC9166168; doi:10.1007/s12603-022-1806-8)

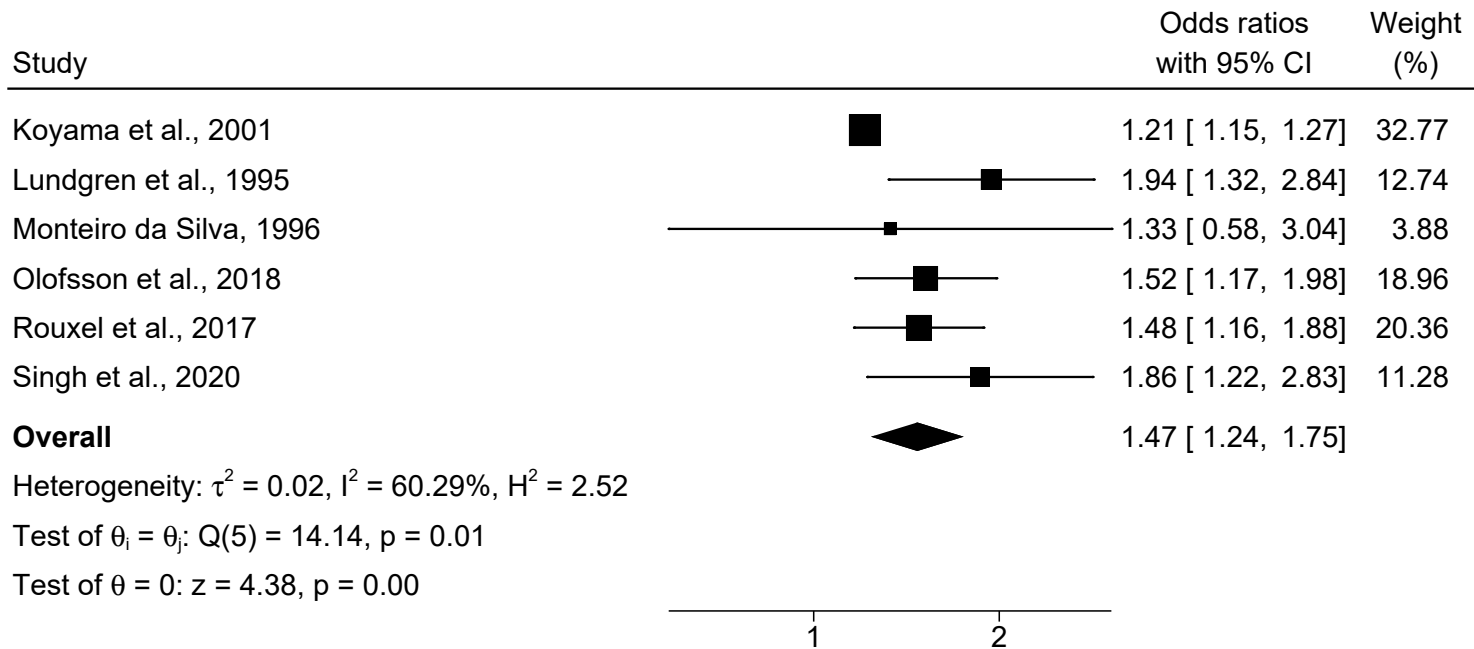

Random-effects REML model

Supplement: Supplementary file 2 — Supplementary material, approximately 95.0 KB. [file 12603_2022_1806_MOESM2_ESM.pdf]

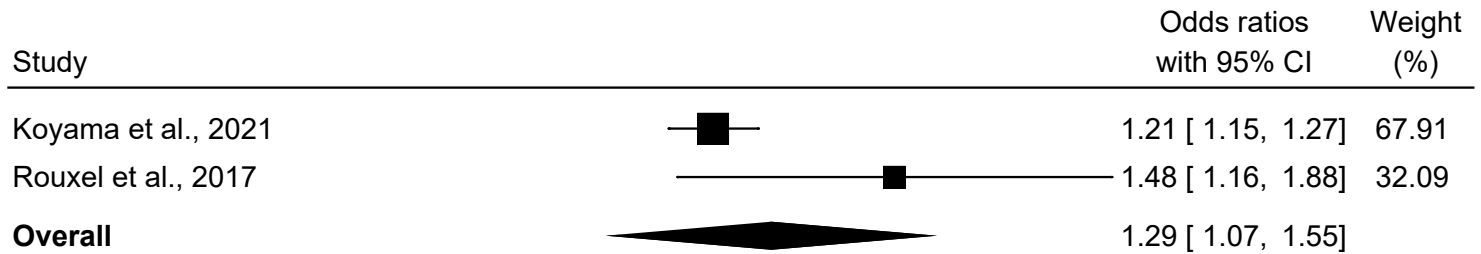

Heterogeneity:  $\tau^2 = 0.01$ ,  $I^2 = 61.02\%$ ,  $H^2 = 2.57$   
Test of  $\theta_i = \theta_j$ :  $Q(1) = 2.57$ ,  $p = 0.11$   
Test of  $\theta = 0$ :  $z = 2.71$ ,  $p = 0.01$

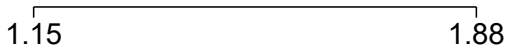

Random-effects REML model

Supplement: Supplementary file 3 — Supplementary material, approximately 92.9 KB. [file 12603_2022_1806_MOESM3_ESM.pdf]
